# Higher-order Network Analysis of Fine Particulate Matter (*PM*_2.5_) Transport in China at City Level

**DOI:** 10.1038/s41598-017-13614-7

**Published:** 2017-10-16

**Authors:** Yufang Wang, Haiyan Wang, Shuhua Chang, Maoxing Liu

**Affiliations:** 1 Department of Statistics, Tianjin University of Finance and Economics, Tianjin, 300222 China; 2 School of Mathematical and Natural Sciences, Arizona State University, AZ, 85069 USA; 3 Coordinated Innovation Center for Computable Modeling in Management Science, Tianjin University of Finance and Economics, Tianjin, 300222 China; 4 Department of Mathematics, North University of China, Shanxi, 030051 China

## Abstract

Specification of *PM*_2.5_ transmission characteristics is important for pollution control and policymaking. We apply higher-order organization of complex networks to identify major potential *PM*_2.5_ contributors and *PM*_2.5_ transport pathways of a network of 189 cities in China. The network we create in this paper consists of major cities in China and contains information on meteorological conditions of wind speed and wind direction, data on geographic distance, mountains, and *PM*_2.5_ concentrations. We aim to reveal *PM*_2.5_ mobility between cities in China. Two major conclusions are revealed through motif analysis of complex networks. First, major potential *PM*_2.5_ pollution contributors are identified for each cluster by one motif, which reflects movements from source to target. Second, transport pathways of *PM*_2.5_ are revealed by another motif, which reflects transmission routes. To our knowledge, this is the first work to apply higher-order network analysis to study *PM*_2.5_ transport.

## Introduction

Accompanying the world’s fastest-growing industrialization and the consequent large amount of vehicle exhaust, China’s increasing occurrences of haze, especially *PM*_2.5_ (particulate matter smaller than 2.5 μm), have been linked to de\creased visibility, negative effects on human health, and influence on global climate. Air pollution has been one of the world’s most important eco-environmental problems. In 2012, a new ambient air quality standard (GB 3095–2012) was set by the Chinese Environmental Protection Agency (EPA), which adds *PM*_2.5_ into the existing list of regularly monitored species. *PM*_2.5_ originates from many sources, such as road dust, vehicle exhaust, biomass burning, industrial emission and agriculture activities, as well as from regionally transported aerosols.

Regionally transported aerosols are an important factor for *PM*_2.5_ pollution^1–6^. There are a number of studies on regional transport for *PM*_2.5_. In^2^, it was found that the air quality of Shanghai is largely influenced by the air masses from the north, east and west directions, accounting for 44.8%, 30.4%, and 24.8% of all the air masses respectively. In^3^, the contribution of regional transport to *PM*_2.5_ was estimated in Lingcheng on the North China Plain. The *PM*_2.5_ from regional transport contributed 31.6% of the *PM*_2.5_ concentrations, with only 15.4% from the local emissions.

It is debatable how far *PM*_2.5_ can spread. A number of research works have studied *PM*_2.5_ transport in local, regional, or long-range scale^1–8^. In the existing works on *PM*_2.5_ transmission, it’s unclear how “local,” “regional,” and “long-range” transport are defined and distinguished. Relative geographic distances are indispensable factors for determining the pollution level. In this paper, if cities are separated by more than a certain distance, we assume their *PM*_2.5_ has no influence on each other.

In addition, the pollution level is highly influenced by meteorological conditions such as wind speed and wind direction^9–14^, which dramatically influence the diffusion, accumulation, and transport of air pollutants^15,16^. Generally, greater wind speed leads to stronger turbulence, resulting in more favorable dispersion conditions for pollutants^17^. Wind direction significantly affects *PM*_2.5_ transport because of the spatial distribution of pollution sources and air pollutants’ transportation^18^.

Mountains between cities are also a major factor influencing *PM*_2.5_ concentration. Where mountains exist, air does not flow between the cities. As depicted in^19^, Beijing is surrounded by mountains in three directions, and polluted air can not be easily expelled in that special geographical environment. Chongqing lies in a mountainous area of China. Influenced by the specific topographic condition, Chongqing is in the region of lowest wind speed over China. In this paper, we considered thirteen major mountains in China to build a city-network, in which the *PM*_2.5_ of any two cities has no reciprocal influence, if there is a mountain between them.

*PM*_2.5_ has significant spatial and temporal characteristics in China^5,20^. Regionally, *PM*_2.5_ concentrations are generally higher in northern regions than in southern regions and tend to be higher in inland regions than in the coastal regions. Seasonally, the level of *PM*_2.5_ is highest in winter and lowest in summer. In wintertime, except for emissions from fossil fuel combustion and biomass burning, meteorological conditions largely contribute to the high concentrations of *PM*_2.5_. More frequent occurrences of stagnant weather, less rainfall, and low temperature are not good for pollution dispersion. Therefore, we choose January of 2016 for this research.

Presently, most of the methods for studying *PM*_2.5_ can be divided into two groups: deterministic and statistical approaches. Deterministic methods^21,22^ mainly focus on the formation mechanism of *PM*_2.5_ from the respective of meteorological-chemistry. In comparison, the statistical approaches, such as linear regression models^23,24^, neural networks^25^, and nonlinear regression models^26,27^, aim to detect certain correlated patterns between air quality data and various selected predictors, thereby predicting the pollutant concentrations in future. Each approach addresses problems from different perspectives.

Network analysis is an important and global method to study relationships between objects^28,29^, that can be organized into a graph. In graph theory, objects are presented as nodes and relationships between two nodes are presented as edges. Network analysis can group nodes into clusters whose members have certain common characteristics. In general, there are more connections between the nodes within a cluster than between the nodes in different clusters. Yang *et al*.^20^ have applied the network tool in studying *PM*_2.5_. In^20^, the correlation between two *PM*_2.5_ emission profiles are investigated, and then network analysis is applied to cluster cities in China. The network structure in their work is depicted at the level of individual nodes and edges, which are considered to be lower-order connectivity patterns of complex networks.

**Figure 1:**
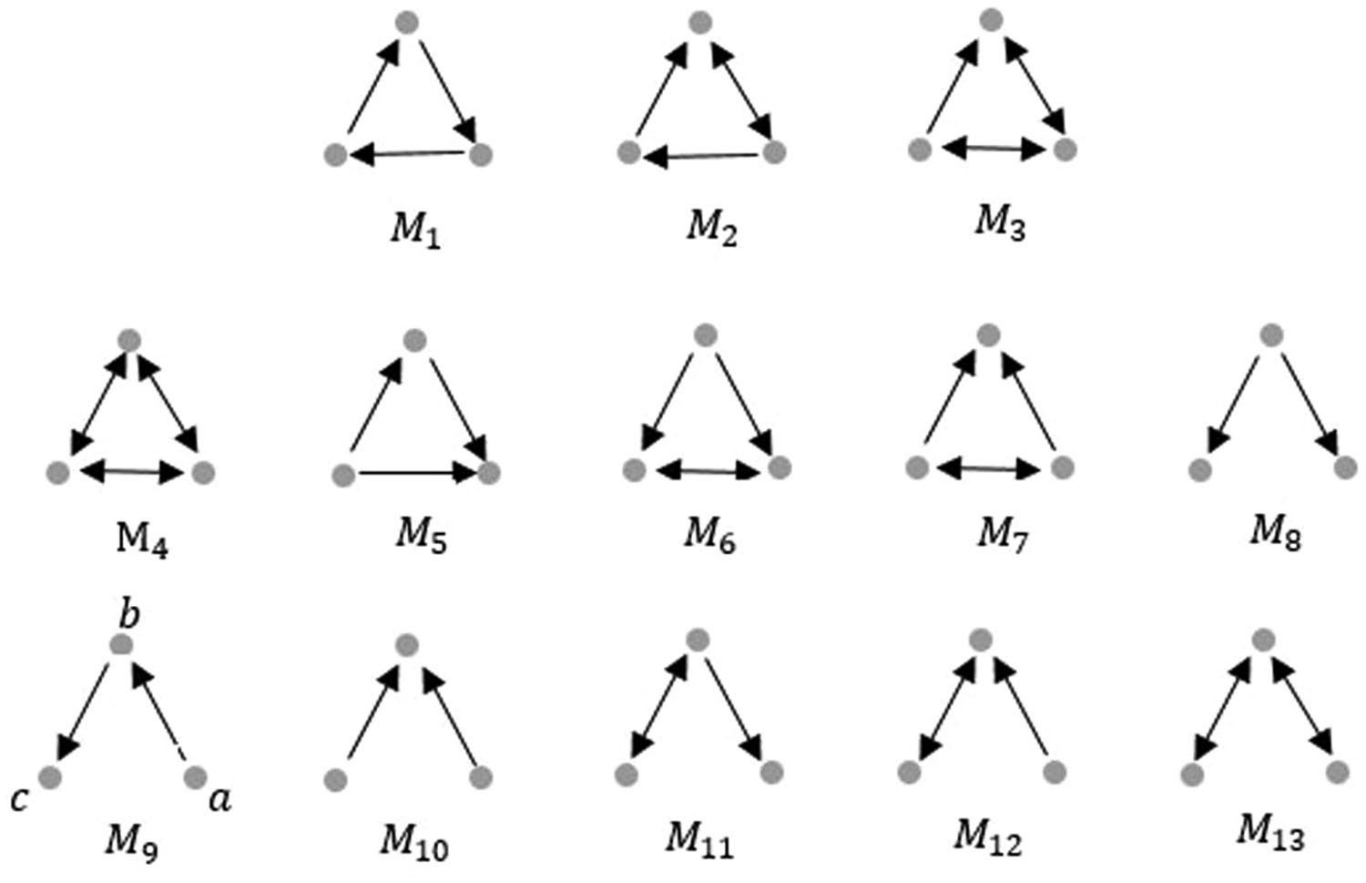
Triangular motifs.

Using higher-order organizations of complex networks as the basic building blocks of complex network can help us understand the fundamental structures of complex systems. The most common higher-order organization of complex networks is network motifs^30,31^. In particular, three-node motifs (Fig. 1) appear frequently in networks. In air traffic patterns, *M*_8_–*M*_13_ are fundamental units of network. *M*_7_–*M*_7_ are structural hubs in the brain. A generalized framework^32^ is developed for clustering networks based on higher-order connectivity patterns. In^32^, different network motifs can result in different higher-order clusters. Motifs (*M*_5_, *M*_6_, or *M*_8_) depict differing hierarchical flow between species in the Florida Bay ecosystem food web.

In this paper, we apply motif-based higher-order organization of complex networks to study *PM*_2.5_ transmission and analyze structures in each city-cluster by motif analysis. Specifically, this paper aims to cluster 189 cities in China and identify major potential *PM*_2.5_ contributors and regional transport pathways in each cluster. We first build an adjacency matrix of the complex network, combining geographic distance, wind speed, wind direction, mountains, and *PM*_2.5_ concentration. Then the cities are clustered by using the motif-based higher-order organization of complex networks. Then, we apply motif analysis to identify the structure in each cluster.

To our best knowledge, this is the first work to apply higher-order organization of complex networks to *PM*_2.5_ transmission. Network analysis not only gives a global view to examine *PM*_2.5_ transmission, but also reveals an internal structure of pollution between cities in China. This research can provide valuable information for the Chinese government to implement air pollution control.

## Results

**Figure 3:**
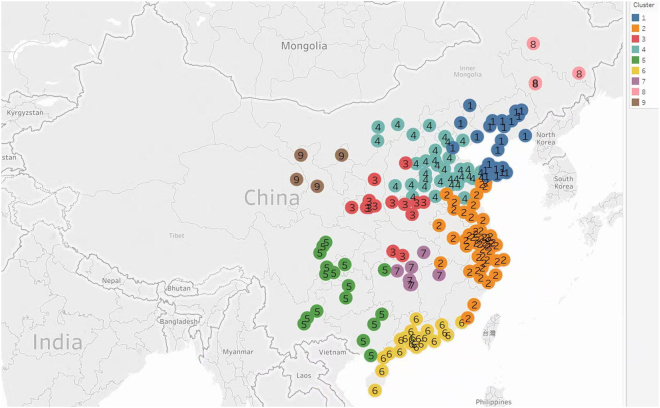
Nine clusters obtained by *m*_8_-motif spectral clustering algorithm. Tableau Public 10.3 (https://public.tableau.com/) was used to create the map.

**Figure 5:**
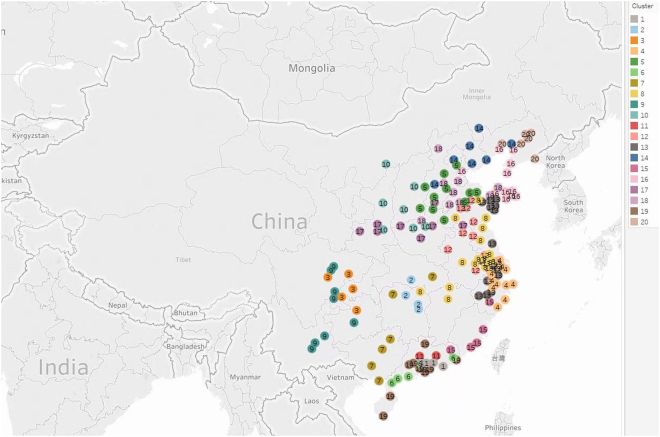
20 clusters obtained by *m*_9_-motif spectral clustering algorithm. Tableau Public 10.3 (https://public.tableau.com/) was used to create the map.

In Figs 3 and 5, a circle with its cluster number represents a city. The cities in a cluster are more densely connected with each other but sparsely connected with the cities in other clusters. In accordance with specific characteristics of *PM*_2.5_ emissions in China, a cluster will usually consist of cities in the same province or close geographical proximity.

### Clustering 189 cities into groups and identifying major potential pollution contributors in each cluster by motif *m*_8_

**Figure 2:**
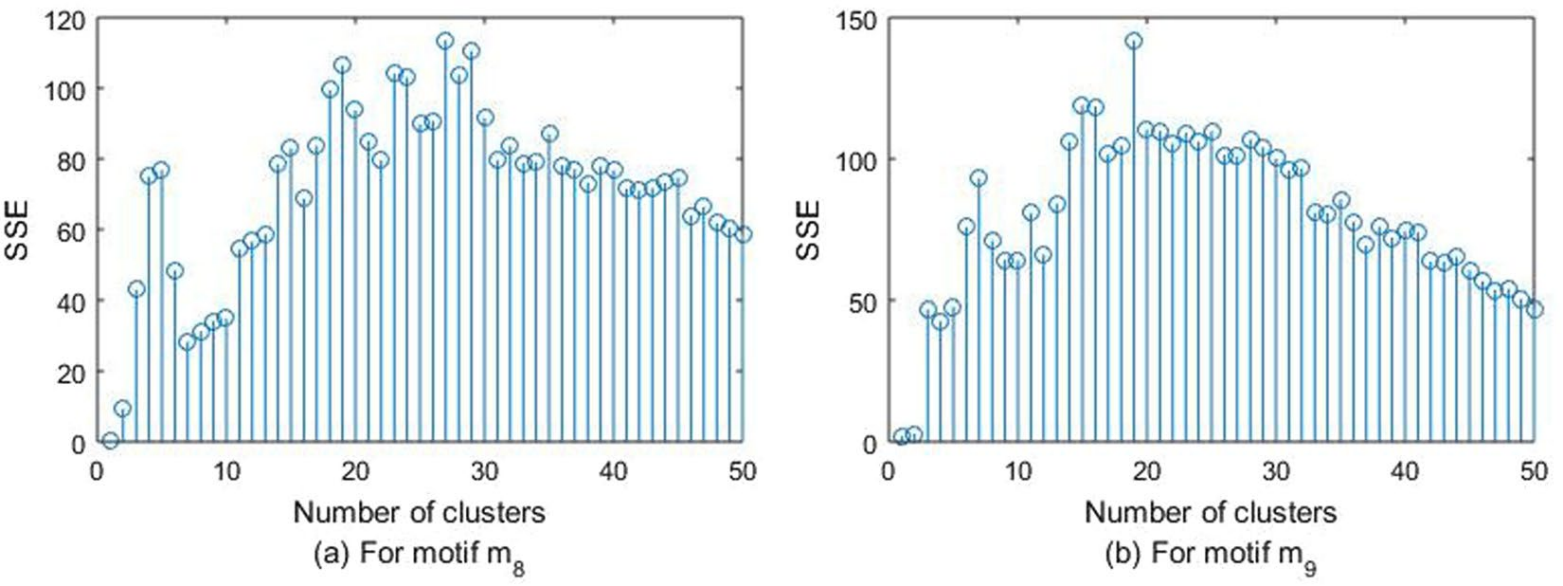
SSE varies with the number of clusters (K).

Motif *m*_8_ is chosen to identify major potential pollution contributors in each cluster. After we perform a higher-order spectral clustering algorithm, three connected components and some isolated points are included (see the Supplementary Table S1) in the *m*8-motif adjacency matrix of 189 cities. The largest connected component contains 170 cities, which form seven clusters. The number of total clusters *K* = 7 makes SSE relatively smaller, which can be seen from Fig. 2(a). Here SSE is defined at the end of this paper. The other two connected components compose cluster 8 (Changchun, Daqing, Jilin, and Mudanjiang) and cluster 9 (Jinchang, Lanzhou, Xining, and Yinchuan) respectively. Thus, nine clusters (see Fig. 3 and Supplementary Table S1) are obtained by a motif *m*_8_-based spectral clustering algorithm. The remaining 11 isolated cities can be explained by their geographic characteristics. Kelamayi, Wulumuqi, and Kuerler are located in the Mongolia Autonomous Region, and Jiayuguan is near the Mongolia Autonomous Region. Lhasa is a plateau area. Qiqihaer and Haerbin are in the most northerly province of China. Two representative clusters, cluster 2 (including Shanghai) and cluster 4 (including Beijing), are illustrated below.

**Figure 4:**
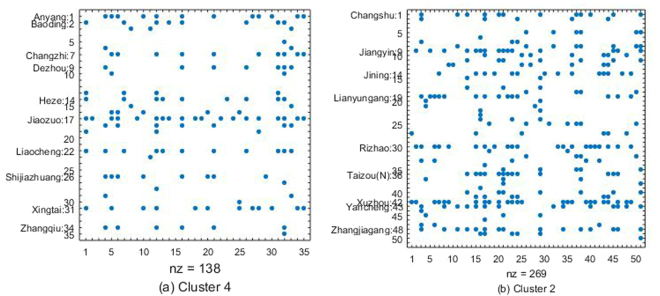
Spy plot of two representative clusters of Fig. 3 in January of 2016. The major potential *PM*_2.5_ contributors in each cluster are marked in the plot. The number order in the spy plot is the ID in Supplementary Table S1.

In cluster 4, there are 31 cities, covering most of northern China. They are shown in Fig. 4(a) and Supplementary Table S1. From the spy plots, we can observe that some cities of y-axis direction correspond to more dots in the horizontal line, which indicates that they have more out-direction arrow lines than other cities in the network subgraph of the cluster, such as Anyang, Baoding, Jiaozuo, Xingtai, and some cities, which are labeled in the spy plot. They are major potential *PM*_2.5_ contributors of Cluster 4. This is in agreement with the results of^5,33^. They concluded that the above cities are the heavily haze-affected cities in Beijing-Tianjin-Hebei, and that pollution from Shandong and Henan provinces by regional transport is also an important factor for the *PM*_2.5_ of North China.

In cluster 2, there are 51 cities, including all the cities from the Yangtze River delta and some of Shandong’s coastal cities, as shown in Fig. 4(b) and Supplementary Table S1. Jining, Xuzhou, Yancheng, Zhangjiagang, and some cities that are labeled in the spy plot are the potential *PM*_2.5_ contributors of cluster 2. Most of the potential *PM*_2.5_ contributors are in the north part of the cluster; this agrees with the spatial characteristics of *PM*_2.5_^5,20^. Note that although Shanghai is a metropolis, it is not a major source of pollution in the cluster. Our conclusion accords with the results of^2^.

### Clustering 189 cities into groups and identifying transport pathways in each cluster by motif *m*_9_

We choose motif *m*_9_ to identify transport pathways in each cluster. We can see, from Fig. 5, that only 166 cities are shown and they are clustered into 20 groups. The remaining 23 cities are isolated and these isolated cities can also be explained by their geographic characteristics. Some of them are from the Mongolia Autonomous Region, the plateau area, the most northerly part of China, or from Gansu and Ningxia. All the clustering results are listed in Supplementary Table S2.

In Fig. 2, SSE is smaller when the number of total clusters *K* = 10. However, clustering with *K* = 10 leads to more cities in each cluster. It’s difficult to see the transport pathways clearly when more cities appear in each cluster. Therefore, we choose *K* = 20 to cluster cities and identify transport pathways in each cluster through motif analysis.

**Figure 6:**
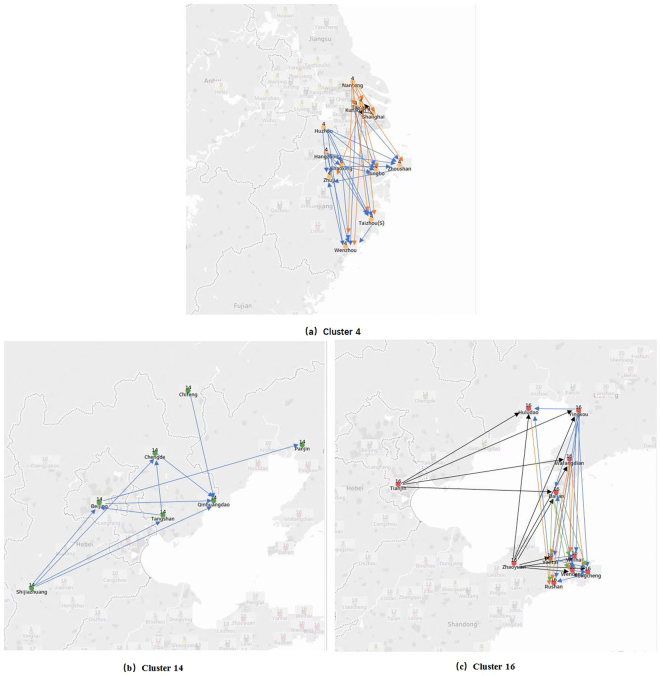
*m*_9_-motif analysis for three representative clusters obtained by motif spectral clustering algorithm based on January of 2016. Tableau Public 10.3 (https://public.tableau.com/) was used to create these maps.

Cluster 4 (including Shanghai), cluster 14 (including Beijing), and cluster 16 (including Tianjin) are shown in Fig. 6. For cluster 4, the *PM*_2.5_ transport pathway originates from Nantong and Huzhou in northwest to Wenzhou, Taizhou(s), Ningbo and Zhoushan in southeast. Shanghai is also generally downwind of the most developed and polluted YRD region in special meteorological conditions, which accords with^2^. For cluster 14, the main *PM*_2.5_ transport pathway is from Shijiazhuang to the northeast of the cluster and the detailed *PM*_2.5_ transport pathway is shown in Fig. 6(b). Shijiazhuang is a key controlling point because of its relative high *PM*_2.5_ concentration and its location upwind of other cities in the cluster. Beijing’s pollution is partly from Shijiazhuang, as described in^5^. Cluster 16 includes Tianjin and cities of Liaotung peninsula, Shangdong peninsula. All of the cities are around Bohai; therefore wind affecting these cities varies frequently and wind directions are not all the same in different cities at the same time. One possible *PM*_2.5_ transport pathway originates from Tianjin, halfway between Huludao, Yingkou,Wafangdian and Dalian in Liaotung peninsula, and arrives at Yantai, Weihai and some cities in Shangdong peninsula. Another possible *PM*_2.5_ transport pathway is from Zhaoyuan to Yantai, Weihai and some cities in Shangdong peninsula, or to Huludao, Yingkou and some cities in Liaotung peninsula.

## Discussion

In this paper, higher-order organization of complex network and spectral clustering methods are used to group cities in China. We obtain two major conclusions: specifically, major potential *PM*_2.5_ contributors and *PM*_2.5_ transport pathways. Clustering of complex networks often provides a global view of the underlying networks. Through the new clustering method, we presents a new framework to investigate the transmission of PM 2.5 among major cities in China.

In general, statistical methods tend to apply data over a long period. The complex network we use in this paper intends to analyze the relationship of nodes in a certain state and reveals the essential structure of a complex system. We intend to use the clustering method to identify the city-network, to aggregate cities, and to identify major potential *PM*_2.5_ contributors and transport pathways. As a result, in this study we collect data only for a short period. Specifically, only January data are used; this is justified for several reasons. *PM*_2.5_’s concentration shows an apparent seasonal pattern. High–frequency and high–concentration *PM*_2.5_ days usually occur in winter^9,34^. This is mainly due to meteorological conditions. In a short period, some meteorological conditions that affects the *PM*_2.5_ can be thought to be relatively stable, and this is helpful for simplifying models. This is the main reason we choose data from one month for our study. As a result, less important factors such as temperatures and atmospheric pressure can be ignored; thus, more important factors can be considered in a relatively simple model. As in^1,9^, we can ignore atmospheric pressure and temperature and consider major meteorological factors such as wind speed and wind direction that influence *PM*_2.5_ concentration in this paper. Wind speed and wind direction vary in each city constantly and they drive air pollution transport between cities. In constructing the adjacency matrix for the network, we choose the monthly prevailing wind direction and monthly average wind speed. This approach allows us to better describe the fact of the frequent change of wind speed and direction in the present study. We believe the data from January suffice for identifying major potential pollution contributors and pollution transport pathways.

We assume that *PM*_2.5_ in city *i* has no influence on city *j*, if the straight-line geographical distance of the two cities is more than 500 kilometers. *PM*_2.5_ flow will dissipate during the propagation. In addition, when the straight-line geographical distance of the two cities is more than 200 kilometers, we assume *PM*_2.5_ in city *i* has influence on city *j*, only if city *i*’s *PM*_2.5_ concentration is higher than city *j*’s at certain extent. We use “500 kilometers” and “200 kilometers” as the dividing values, mainly inspired by^9^, which concluded that aerosol nucleation and growth processes occur on the regional (several hundred kilometers) to urban (less than 100 kilometers) scales. Although there are many research works on regional transport of *PM*_2.5_, it is an open question as to how far *PM*_2.5_ can travel. In addition, we believe that many physical, biological and social models, for example^35–44^, could be used for estimating/predicting the long range transport of *PM*_2.5_.

Because meteorological conditions are complex, additional factors affecting *PM*_2.5_ should be considered in future studies. In addition, *PM*_2.5_ in city *i* has influence on other cities and the incidence should be inversely proportional to the geographic distance. Therefore, it is more important that weighted complex networks should be considered in future.

In this paper, we consider some meteorological conditions and geographical data to cluster cities in China and identify the inner structure of each cluster. However, *PM*_2.5_ transmission between cities is a very complex issue. Economy, population, in-vehicle commuting, and many others are also indispensable factors that influence *PM*_2.5_ transmission. More economic factors and social factors will be considered in our future work.

## Data and Methods

### Data

In this paper, we focus on the top 189 pollution–monitoring cities in China’s mainland, which cover all 34 provincial-level regions of China. The most polluted and the major cities are all included, such as Beijing, Shanghai and Guangzhou.

Data from January 2016 are used in this work to identify major *PM*_2.5_ pollution contributors and transport pathways in each cluster. The data that we collect in this paper are as follows: (1) *PM*_2.5_ monthly average concentration is calculated based on ground air quality monitoring data from China’s National Environmental Monitoring. (2) The geo-location information in the forms of latitude and longitude of 189 cities are from Google Earth. (3) Thirteen major mountains with high altitudes in China (see Supplementary Table S3) are included in this paper. (4) Wind speed and wind direction data is from the China Meteorological Administration. Wind directions are classified into eight directions (e.g., N, E, S, W, W-S, E-S, W-N, E-N), We use the monthly prevailing wind direction of each city in January. The scaling of wind speed is based on the Jenks Natural Breaks Classification method^10^. Wind speed(ws) is divided into eight levels: $ws\le 0.7m/s$ (Level-1), $\mathrm{0.7 < }ws\le 1.1m/s$ (Level-2), $1.1 < ws\le 1.6m/s$ (Level-3), $\mathrm{1.6 < }ws\le 2.1m/s$ (Level-4), $\mathrm{2.1 < }ws\le 2.7m/s$ (Level-5), $\mathrm{2.7 < }ws\le 3.4m/s$ (Level-6), $\mathrm{3.4 < }ws\le 4.4m/s$ (Level-7) and $ws\mathrm{ > 4.4}m/s$ (Level-8). We use the monthly average wind speed. Here “monthly average” means the arithmetic average of the mean concentration levels or mean wind speed of each day in a calendar month.

### Motif-based higher-order spectral clustering algorithm

The motif-based higher-order spectral clustering algorithm in the supplementary materials of^32^ unifies motif analysis^30^ and k-means spectral clustering^45^ to reveal new organizational patterns and modules in complex systems. We use the method to cluster 189 cities in China and identify major potential *PM*_2.5_ contributors and *PM*_2.5_ transport pathways in clusters. The major steps are listed below.

1. Building the adjacency matrix *A* of the network and choosing motif *M* of interest. Specifically, in this paper, matrix *A* is built as follows: ${m}_{8}$ and ${m}_{9}$ are chosen as the building modules to reveal the essential structures of the complex network; ${m}_{8}$ reflects reveal the relationship between source and victims; ${m}_{9}$ reflects the transmission route.
2. Computing the motif adjacency matrix ${W}_{M}$, whose entry ${W}_{M}(i,j)$ equals the number of the motif instances of motif *M* with node *i* and node *j*.
3. Clustering 189 cities by spectral clustering algorithm through the motif adjacency matrix ${W}_{M}$.
  - Computing the normalized motif Laplacian ${L}_{M}=I-{D}_{M}^{-\mathrm{1/2}}{W}_{M}{D}_{M}^{-\mathrm{1/2}}$, where ${D}_{M}$ is diagonal matrix with ${({D}_{M})}_{ii}={\sum }_{j}{({W}_{M})}_{ij}$.
  - Forming matrix $X$, st.$X=[{x}_{1},{x}_{2},\cdots ,{x}_{k}]$, where ${x}_{1},{x}_{2},\cdots ,{x}_{k}$ are the k largest eigenvectors of ${L}_{M}$.
  - Calculating matrix *Y*, whose entry is ${Y}_{ij}={X}_{ij}/({\sum }_{j}{X}_{ij}^{2}{)}^{\mathrm{1/2}}$.
  - Taking each row of *Y* as a point in ${R}^{k}$ and cluster the points into k clusters via k-means method^45^. In this paper, the optimal number of cluster(K) is chosen as follows, which is inspired by^46^.
  - City *j* is assigned to cluster *j* if and only if row *j* of matrix *Y* is assigned to cluster *j*.
4. Analyzing every cluster using the motifs of (1). This paper applies motif ${m}_{8}$ to analyze major potential contributors. In the social network graph, a node with the largest numbers of edges is commonly considered as source, from which information begins to disperse^47^. After using motif ${m}_{8}$ to cluster 189 cities in the complex network, a city with more out-direction arrow lines shows that it has relatively high *PM*_2.5_ concentration and it has a high influence ratio on the other cities of the cluster. The city can be regarded as one major *PM*_2.5_ pollution contributor in the cluster. This can be seen through the spy plot, which illustrates the network structure of the cluster. Motif ${m}_{9}$ helps find *PM*_2.5_ transport pathway in every cluster. In Fig. 1, ${m}_{9}$ corresponds to the *PM*_2.5_ flow from city a to city b, then from city b to city c.

#### Building an Adjacency Matrix

A network can be represented as a matrix, which is called the sociomatrix^44^ or adjacency matrix. Suppose the number of nodes is n. Let V and E be the sets of nodes and edges in the network, respectively. Then the adjacency matrix of the network can be expressed by matrix A∈{0, 1}_*nn*_. An entry ${A}_{ij}\in \mathrm{\{0},\mathrm{1\}}$ denotes whether there is a link between node ${v}_{i}$ and node ${v}_{j}$. If node ${v}_{i}$ and node ${v}_{j}$ are adjacent, then ${A}_{ij}=1$. Otherwise, ${A}_{ij}=0$. If the network is undirected, the adjacency matrix A is symmetric. However, in some situations, interactions between two different individuals are directional. In Twitter, for example, one user x follows another user *y*, but user *y* does not necessarily follow user *x*. In this case, the follower- followee network is directed and asymmetrical.

Based on *PM*_2.5_ monthly-average concentration, geographic distance between cities, monthly prevailing wind direction, monthly average wind speed, and mountains between cities (189 cities in China in January 2016), the detailed procedure for building the adjacency matrix is as follows:

(1) Adjacency matrix based on distance (A_1_): Based on the latitudes and longitudes of 189 cities, the relative geographic distances are calculated. The entry ${A}_{1}(i,j)=0$, if the relative geographic distance is more than 500 kilometers. Otherwise, ${A}_{1}(i,j)=1$. The assumption is plausible because *PM*_2.5_ of each city has no effect on another city, if they are distant from each other.

We choose 500 kilometers, because it is an empirical value through numerical simulation. This is in agreement with^9^, which found that aerosol nucleation and growth processes occur on the regional (several hundred kilometers) to urban (less than 100 kilometers) scales.

(2) Adjacency matrix based on mountain (A_2_): In the planimetric map, a major mountain can be expressed by a line segment through its latitudes and longitudes, which is called a mountain-segment. Therefore the 13 major mountains we considered are depicted by 13 different line segments. Meanwhile, there is a line segment between any two cities, which is called a cities-segment. If the cities-segment between city *i* and city *j* has a cross point with any of the 13 mountain-segments, the entry ${A}_{2}(i,j)=0$. Otherwise, ${A}_{2}(i,j)=1$.

(3) Adjacency matrix based on wind (A_3_): Wind speed and wind direction jointly affect the propagation of *PM*_2.5_ flow. Due to the wind direction, the effect from wind on *PM*_2.5_ transmission is directional. Specifically, *PM*_2.5_ of city *i* may flow to city *j*. But it is possible that *PM*_2.5_ of city *j* may not be blown to city *i*.

We assume city *i*’s *PM*_2.5_ has no effect on any other cities, if wind level is less than 2. When the speed level is more than 2 (more than 1.1 m/s), the wind direction is a key point, determining whether *PM*_2.5_ flowing from city *i* affects city *j*. Specifically, in the planimetric map, there is a directional line segment from city *i* to city *j*. If the angle ${\theta }_{ij}$, between city *i*’s wind direction and the directional line segment from city *i* to city *j*, is less than 90 degree, we think city *i*’s wind can flow to city *j*.

The overall effect of wind from city i to city j is calculated by ${A}_{3}(i,j)={w}_{i}cos({\theta }_{ij})$, where ${w}_{i}$ is the wind speed of city i.

(4) Adjacency matrix based on *PM*_2.5_ (A_4_): The paper aims to study the major pollution contributors and the pollution transport pathways. And we are particularly interested in that how a city with high *PM*_2.5_ concentration affects a city with low concentration. Specifically, two situations are considered below.

Situation One: When the geographic distance is less than 200 kilometers, the *PM*_2.5_ of city i has effect on city *j*, as long as the *PM*_2.5_ concentration of it is higher than city *j*’s, then ${A}_{4}(i,j)=1$. Otherwise, ${A}_{4}(i,j)=0$.

Situation Two: *PM*_2.5_ flow will dissipate during the propagation. Therefore, when the geographic distance is more than 200 kilometers, if and only if city *i*’s *PM*_2.5_ concentration is *α* × *d*_ij_ higher than city *j*’s, then ${A}_{4}(i,j)=1$. Otherwise, ${A}_{4}(i,j)=0$. Here *d*_ij_ is the geographic distance between city *i* and city *j* and $\alpha =0.01$ is an empirical threshold value through numerical simulation. Better α should be considered in future work according to the meteorological condition. *α* × *d*_ij_ is a degree of *PM*_2.5_ concentration, increasing with the geographic distance d_ij_.

Clustering and motif analysis are based on the adjacency matrix $A={A}_{1}\circ {A}_{2}\circ {A}_{3}\circ {A}_{4}$, where “$\circ$” is the Hadamard (entry-wise) product. Namely, *PM*_2.5_’s propagation is the combined effects of geographic distance, mountain, wind and *PM*_2.5_ concentration.

#### Selecting K

As in^46^, the sum of the squared distance between each member of a cluster and its cluster centroid (SSE) is defined as

$$SSE=\sum _{i\mathrm{=1}}^{K}\sum _{x\in {C}_{i}}dist{({c}_{i},x)}^{2},$$

where *x* is a city; *c*_i_ is the centroid of cluster *C*_i_; *C*_i_ is the i th cluster (cluster *i*); dist is the the standard Euclidean distance between two cities of Euclidean space. *K* is the number of clusters, and the optimal value is chosen from 2 to 50, which makes dist smallest. The *K* larger than 50 has not much meaning for clustering 189 cities, which leads to too-detailed clustering.

## Electronic supplementary material

SUPPLEMENTARY INFORMATION

## References

[1] Zheng G (2015). Exploring the severe winter haze in Beijing: the impact of synoptic weather, regional transport and heterogeneous reactions. Atmospheric Chemistry and Physics.

[2] Wang H (2016). Chemical composition of pm 2.5 and meteorological impact among three years in urban Shanghai, China. Journal of Cleaner Production.

[3] Chen D (2017). Estimating the contribution of regional transport to pm 2.5 air pollution in a rural area on the North China plain. Science of The Total Environment.

[4] Xiong Y, Zhou J, Schauer JJ, Yu W, Hu Y (2017). Seasonal and spatial differences in source contributions to pm 2.5 in Wuhan, China. Science of the Total Environment.

[5] Zhang YL, Cao F (2015). Fine particulate matter (pm2.5) in China at a city level. Scientific Reports.

[6] Liu J (2014). Source apportionment using radiocarbon and organic tracers forpm2. 5 carbonaceous aerosols in Guangzhou, south China: Contrasting local-and regional-scale haze events. Environmental science & technology.

[7] Baker J (2010). A cluster analysis of long range air transport pathways and associated pollutant concentrations within the UK. Atmospheric Environment.

[8] Saliba NA, Kouyoumdjian H, Roumié M (2007). Effect of local and long-range transport emissions on the elemental composition of pm 10–2.5 and pm 2.5 in Beirut. Atmospheric Environment.

[9] Guo S (2014). Elucidating severe urban haze formation in China. Proceedings of the National Academy of Sciences.

[10] Zhang, B. *et al*. Influences of wind and precipitation on different-sized particulate matter concentrations (pm2.5, pm10, pm2.5–10). Meteorology and Atmospheric Physics 1–10 (2017).

[11] Adams H, Nieuwenhuijsen M, Colvile R (2001). Determinants of fine particle (pm 2.5) personal exposure levels in transport microenvironments, London, UK. Atmospheric Environment.

[12] Guerra, S. *et al*. Effects of wind direction on pm10 and pm2. 5 concentrations in southeast Kansas. Proceedings of the Air & Waste Management Association (2004). 10.1080/10473289.2006.10464559 17117737

[13] Nguyen M-V, Park G-H, Lee B-K (2017). Correlation analysis of size-resolved airborne particulate matter with classified meteorological conditions. Meteorology and Atmospheric Physics.

[14] Westervelt D (2016). Quantifying pm 2.5-meteorology sensitivities in a global climate model. Atmospheric Environment.

[15] Jacob DJ (2009). & Winner, D. A. Effect of climate change on air quality. Atmospheric Environment.

[16] Pearce JL, Beringer J, Nicholls N, Hyndman RJ, Tapper NJ (2011). Quantifying the influence of local meteorology on air quality using generalized additive models. Atmospheric Environment.

[17] Tian G, Qiao Z, Xu X (2014). Characteristics of particulate matter (pm 10) and its relationship with meteorological factors during 2001–2012 in Beijing. Environmental Pollution.

[18] Zhou W, Tie X, Zhou G, Liang P (2015). Possible effects of climate change of wind on aerosol variation during winter in Shanghai, China. Particuology.

[19] Yang F (2011). Characteristics of pm 2.5 speciation in representative megacities and across China. Atmospheric Chemistry and Physics.

[20] Yan S, Wu G (2016). Network analysis of fine particulate matter (pm2. 5) emissions in China. Scientific Reports.

[21] Chuang M-T, Zhang Y, Kang D (2011). Application of wrf/chem-madrid for real-time air quality forecasting over the southeastern United States. Atmospheric Environment.

[22] Yahya K, Zhang Y, Vukovich JM (2014). Real-time air quality forecasting over the southeastern united states using wrf/chem-madrid: Multiple-year assessment and sensitivity studies. Atmospheric Environment.

[23] Li C, Hsu NC, Tsay S-C (2011). A study on the potential applications of satellite data in air quality monitoring and forecasting. Atmospheric Environment.

[24] Benas N, Beloconi A, Chrysoulakis N (2013). Estimation of urban pm10 concentration, based on modis and meris/aatsr synergistic observations. Atmospheric environment.

[25] Mao, X., Shen, T. & Feng, X. Prediction of hourly ground-level pm 2.5 concentrations 3 days in advance using neural networks with satellite data in eastern China. Atmospheric Pollution Research https://doi.org/10.1016/j.apr.2017.04.002 (2017).

[26] Emili E (2010). Pm 10 remote sensing from geostationary seviri and polar-orbiting modis sensors over the complex terrain of the european alpine region. Remote sensing of environment.

[27] Tian J, Chen D (2010). A semi-empirical model for predicting hourly ground-level fine particulate matter (pm 2.5) concentration in southern ontario from satellite remote sensing and ground-based meteorological measurements. Remote Sensing of Environment.

[28] Rosvall M, Esquivel AV, Lancichinetti A, West JD, Lambiotte R (2014). Memory in network flows and its effects on spreading dynamics and community detection. Nature communications.

[29] Leskovec J, Lang KJ, Dasgupta A, Mahoney MW (2009). Community structure in large networks: Natural cluster sizes and the absence of large well-defined clusters. Internet Mathematics.

[30] Milo R (2002). Network motifs: simple building blocks of complex networks. Science.

[31] Yavero ğlu ÖN (2014). Revealing the hidden language of complex networks. Scientific Reports.

[32] Benson AR, Gleich DF, Leskovec J (2016). Higher-order organization of complex networks. Science.

[33] Huang R-J (2014). High secondary aerosol contribution to particulate pollution during haze events in China. Nature.

[34] Yang J (2015). Concentrations and seasonal variation of ambientpm2.5 and associated metals at a typical residential area in Beijing, China. Bulletin of environmental contamination and toxicology.

[35] Chen MH, Wang L, Sun SW, Wang J, Xia CY (2016). Evolution of cooperation in the spatial public goods game with adaptive reputation assortment. Physics Letters A.

[36] Chen MH, Wang L, Wang J, Sun SW, Xia CY (2015). Impact of individual response strategy on the spatial public goods game within mobile agents. Applied Mathematics and Computation.

[37] Xia CY, Miao Q, Wang J, Ding S (2014). Evolution of cooperation in the traveler’s dilemma game on two coupled lattices. Applied Mathematics and Computation.

[38] Sun GQ, Wang CH, Wu ZY (2017). Pattern dynamics of a Gierer–Meinhardt model with spatial effects. Nonlinear Dynamics.

[39] Sun GQ, Wang SL, Ren Q, Jin Z, Wu YP (2015). Effects of time delay and space on herbivore dynamics: linking inducible defenses of plants to herbivore outbreak. Scientific Reports.

[40] Li L (2015). Patch invasion in a spatial epidemic model. Applied Mathematics and Computation.

[41] Sun GQ (2017). Transmission dynamics of cholera: Mathematical modeling and control strategies. Communications in Nonlinear Science and Numerical Simulation.

[42] Li L (2016). Monthly Periodic Outbreak Of Hemorrhagic Fever With Renal Syndrome In China. Journal of Biological Systems.

[43] Sun GQ, Jusup M, Jin Z, Wang Y, Wang Z (2016). Pattern transitions in spatial epidemics: Mechanisms and emergent properties. Physics of Life Reviews.

[44] Wasserman, S. & Faust, K. Social network analysis: Methods and applications, vol. 8 (Cambridge university press, 1994).

[45] Ng, A. Y., Jordan, M. I. & Weiss, Y. On spectral clustering: Analysis and an algorithm. In Advances in neural information processing systems, 849–856 (2002).

[46] Baxter LK, Sacks JD (2014). Clustering cities with similar fine particulate matter exposure characteristics based on residential infiltration and in-vehicle commuting factors. Science of the Total Environment.

[47] Valente TW (2012). Network interventions. Science.

