# Supplementary material for: Higher-order Network Analysis of Fine Particulate Matter (PM2.5) Transport in China at City Level
Source: Sci Rep. 2017 Oct 16;7:13236. doi: 10.1038/s41598-017-13614-7 (PMC5643331; doi:10.1038/s41598-017-13614-7)
Supplement: Supplementary file 1 — SUPPLEMENTARY INFORMATION [file 41598_2017_13614_MOESM1_ESM.pdf]

SUPPLEMENTARY INFORMATION
for

Higher-order Network Analysis of Fine Particulate Matter ($PM_{2.5}$) Transport in China at City Level

YUFANG WANG[1,3,*], HAIYAN WANG[2], SHUHUA CHANG[3,*], and MAOXING LIU[4]

[1]Department of Statistics, Tianjin University of Finance and Economics, Tianjin 300222, China
[2]School of Mathematical and Natural Sciences, Arizona State University, AZ 85069, USA
[3]Coordinated Innovation Center for Computable Modeling in Management Science, Tianjin University of Finance and Economics, Tianjin 300222, China
[4]Department of Mathematics, North University of China, Shanxi 030051, China
[*]shuhua55@126.com, wangyufangminshan@163.com

Table S1: 189 cities (9 clusters) by m_8-motif spectral clustering algorithm based on data in January of 2016.

City	Cluster	ID	City	Cluster	ID	City	Cluster	ID
Anshan	1	1	Wuxi	2	41	Deyang	5	4
Benxi	1	2	Xuzhou	2	42	Guilin	5	5
Chengde	1	3	Yancheng	2	43	Guiyang	5	6
Chifeng	1	4	Yangzhou	2	44	Kunming	5	7
Dalian	1	5	Yiwu	2	45	Liuzhou	5	8
Dandong	1	6	Yixing	2	46	Luzhou	5	9
Fushun	1	7	Zaozhuang	2	47	Mianyang	5	10
Huludao	1	8	Zhangjiagang	2	48	Nanning	5	11
Jinzhou	1	9	Zhenjiang	2	49	Qujing	5	12
Laixi	1	10	Zhoushan	2	50	Yibin	5	13
Panjin	1	11	Zhuji	2	51	Yuxi	5	14
Penglai	1	12	Baoji	3	1	Zhangjiajie	5	15
Qinhuangdao	1	13	Jingzhou	3	2	Zigong	5	16
Rongcheng	1	14	Kaifeng	3	3	Zunyi	5	17
Rushan	1	15	Luoyang	3	4	Dongguan	6	1
Shenyang	1	16	Pingdingshan	3	5	Foshan	6	2
Tianjin	1	17	Sanmenxia	3	6	Guangzhou	6	3
Wafangdian	1	18	Taiyuan	3	7	Haikou	6	4
Weihai	1	19	Tongchuan	3	8	Heyuan	6	5
Wendeng	1	20	Weinan	3	9	Huizhou	6	6
Yantai	1	21	Xian	3	10	Jiangmen	6	7
Yingkou	1	22	Xianyang	3	11	Jieyang	6	8
Zhaoyuan	1	23	Yanan	3	12	Maoming	6	9
Changshu	2	1	Yichang	3	13	Meizhou	6	10
Changzhou	2	2	Zhengzhou	3	14	Qingyuan	6	11
Fuyang	2	3	Anyang	4	1	Sanya	6	12
Fuzhou	2	4	Baoding	4	2	Shantou	6	13
Hangzhou	2	5	Baotou	4	3	Shanwei	6	14
Hefei	2	6	Beijing	4	4	Shaoguan	6	15
Huaian	2	7	Binzhou	4	5	Shenzhen	6	16
Huzhou	2	8	Cangzhou	4	6	Xiamen	6	17
Jiangyin	2	9	Changzhi	4	7	Yangjiang	6	18
Jiaozhou	2	10	Datong	4	8	Yunfu	6	19
Jiaxing	2	11	Dezhou	4	9	Zhanjiang	6	20
Jimo	2	12	Dongying	4	10	Zhaoqing	6	21
Jinhua	2	13	Eerduosi	4	11	Zhongshan	6	22
Jining	2	14	Handan	4	12	Zhuhai	6	23
Jintan	2	15	Hengshui	4	13	Changde	7	1
Jiujiang	2	16	Heze	4	14	Changsha	7	2
Jurong	2	17	Huhehaote	4	15	Nanchang	7	3
Kunshan	2	18	Jiaonan	4	16	Wuhan	7	4
Lianyungang	2	19	Jiaozuo	4	17	Xiangtan	7	5
Linan	2	20	Jinan	4	18	Yueyang	7	6
Lishui	2	21	Laiwu	4	19	Zhuzhou	7	7
Liyang	2	22	Laizhou	4	20	Changchun	8	1
Maanshan	2	23	Langfang	4	21	Daqing	8	2
Nanjing	2	24	Liaocheng	4	22	Jilin	8	3
Nantong	2	25	Linfen	4	23	Mudanjiang	8	4
Ningbo	2	26	Linyi	4	24	Jinchang	9	1
Qingdao	2	27	Pingdu	4	25	Lanzhou	9	2
Quanzhou	2	28	Shijiazhuang	4	26	Xining	9	3
Quzhou	2	29	Shouguang	4	27	Yinchuan	9	4
Rizhao	2	30	Taian	4	28	Kelamayi	isolated	
Shanghai	2	31	Tangshan	4	29	Wulumuqi	isolated	
Shaoxing	2	32	Weifang	4	30	Kuerler	isolated	
Suqian	2	33	Xingtai	4	31	Lasa	isolated	
Suzhou	2	34	Yangquan	4	32	Qiqihaer	isolated	
Taicang	2	35	Zhangjiakou	4	33	Haerbin	isolated	
Taizhou(N)	2	36	Zhangqiu	4	34	Jiayuguan	isolated	
Taizhou(S)	2	37	Zibo	4	35	Nanchong	isolated	
Wenzhou	2	38	Beihai	5	1	Panzhihua	isolated	
Wuhu	2	39	Chengdu	5	2	Shizuishan	isolated	
Wujiang	2	40	Chongqing	5	3	Chaozhou	isolated	

Table S2: 189 cities(20 clusters) by m_9-motif spectral clustering algorithm based on data in January of 2016.

City	Cluster	ID	City	Cluster	ID	City	Cluster	ID
Dongguan	1	1	Zhenjiang	8	16	Tianjin	16	5
Guangzhou	1	2	Deyang	9	1	Wafangdian	16	6
Huizhou	1	3	Guiyang	9	2	Weihai	16	7
Shanwei	1	4	Kunming	9	3	Wendeng	16	8
Changde	2	1	Mianyang	9	4	Yantai	16	9
Changsha	2	2	Qujing	9	5	Yingkou	16	10
Jingzhou	2	3	Yibin	9	6	Zhaoyuan	16	11
Xiangtan	2	4	Yuxi	9	7	Baoji	17	1
Chaozhou	2	5	Zigong	9	8	Handan	17	2
Chengdu	3	1	Eerduosi	10	1	Kaifeng	17	3
Chongqing	3	2	Linfen	10	2	Pingdingshan	17	4
Luzhou	3	3	Luoyang	10	3	Sanmenxia	17	5
Nanchong	3	4	Taiyuan	10	4	Tongchuan	17	6
Zunyi	3	5	Weinan	10	5	Xian	17	7
Hangzhou	4	1	Yanan	10	6	Zaozhuang	17	8
Huzhou	4	2	Zhengzhou	10	7	Cangzhou	18	1
Kunshan	4	3	Heyuan	11	1	Dezhou	18	2
Nantong	4	4	Qingyuan	11	2	Jiaonan	18	3
Ningbo	4	5	Fuyang	12	3	Jiaozuo	18	4
Shanghai	4	6	Laiwu	12	4	Jinan	18	5
Shaoxing	4	7	Linyi	12	5	Laizhou	18	6
Taicang	4	8	Suqian	12	6	Liaocheng	18	7
Taizhou(S)	4	9	Taian	12	7	Penglai	18	8
Wenzhou	4	10	Wuhu	12	8	Zhangjiakou	18	9
Zhoushan	4	11	Xuzhou	12	9	Haikou	19	1
Zhuji	4	12	Yangzhou	12	10	Jiangmen	19	2
Anyang	5	1	Zibo	12	11	Sanya	19	3
Baoding	5	2	Changshu	13	1	Shantou	19	4
Binzhou	5	3	Jiaozhou	13	2	Shaoguan	19	5
Changzhi	5	4	Jiaxing	13	3	Shenzhen	19	6
Dongying	5	5	Jimo	13	4	Yunfu	19	7
Hengshui	5	6	Jinhua	13	5	Zhaoqing	19	8
Heze	5	7	Jintan	13	6	Zhongshan	19	9
Langfang	5	8	Jurong	13	7	Zhuhai	19	10
Xingtai	5	9	Laixi	13	8	Anshan	20	1
Yangquan	5	10	Linan	13	9	Benxi	20	2
Zhangqiu	5	11	Pingdu	13	10	Dandong	20	3
Foshan	6	1	Qingdao	13	11	Fushun	20	4
Jieyang	6	2	Quzhou	13	12	Jinzhou	20	5
Maoming	6	3	Suzhou	13	13	Shenyang	20	6
Yangjiang	6	4	Weifang	13	14	Baotou	isolated	
Zhanjiang	6	5	Wujiang	13	15	Changchun	isolated	
Beihai	7	1	Wuxi	13	16	Daqing	isolated	
Guilin	7	2	Yancheng	13	17	Datong	isolated	
Liuzhou	7	3	Yiwu	13	18	Haerbin	isolated	
Nanning	7	4	Yixing	13	19	Huhehaote	isolated	
Wuhan	7	5	Zhangjiagang	13	20	Jiayuguan	isolated	
Zhangjiajie	7	6	Beijing	14	1	Jilin	isolated	
Changzhou	8	1	Chengde	14	2	Jinchang	isolated	
Hefei	8	2	Chifeng	14	3	Kelamayi	isolated	
Huaian	8	3	Panjin	14	4	Kuerle	isolated	
Jiangyin	8	4	Qinhuangdao	14	5	Lanzhou	isolated	
Jining	8	5	Shijiazhuang	14	6	Lasa	isolated	
Jiujiang	8	6	Tangshan	14	7	Mudanjiang	isolated	
Lianyungang	8	7	Fuzhou	15	1	Panzhihua	isolated	
Liyang	8	8	Lishui	15	2	Qiqihaer	isolated	
Maanshan	8	9	Meizhou	15	3	Shizuishan	isolated	
Nanchang	8	10	Quanzhou	15	4	Wulumuqi	isolated	
Nanjing	8	11	Xiamen	15	5	Xianyang	isolated	
Rizhao	8	12	Dalian	16	1	Xining	isolated	
Shouguang	8	13	Huludao	16	2	Yichang	isolated	
Taizhou(N)	8	14	Rongcheng	16	3	Yinchuan	isolated	
Yueyang	8	15	Rushan	16	4	Zhuzhou	isolated	

Table S3: Thirteen major mountains in China.

Mountain
DaXinAnLing
ChangbaiShan
TaiHangShan
HeLanShan
QiLianShan
QinLing
WuShan
DaBieShan
HengDuanShan
WuYiShan
NanLing
TianShan
GangDiSiShan
